# Electrophysiological Correlates of Basic and Higher Order Cognitive and Affective Theory of Mind Processing in Emerging and Early Adulthood—An Explorative Event-Related Potentials Study to Investigate First-, Second-, and Third-Order Theory of Mind Processing Based on Visual Cues

**DOI:** 10.3389/fnhum.2020.00079

**Published:** 2020-03-31

**Authors:** Benjamin Tesar, Matthias Deckert, Michaela Schmoeger, Ulrike Willinger

**Affiliations:** Department of Neurology, Medical University of Vienna, Vienna, Austria

**Keywords:** cognitive theory of mind, affective theory of mind, event-related potentials, higher order theory of mind, third-order theory of mind, exact low-resolution brain electromagnetic tomography analyses (eLORETA), high performers

## Abstract

Attributing mental states to others in social interactions [Theory of Mind (ToM)] often depends on visual social cues like eye gaze or mimic. This study presents an event-related potentials task (*Brainy-ERP*) that was developed in order to investigate the electrophysiological correlates of first-, second-, and third-order cognitive and affective ToM processing. The task was based on social visual cues and involved electroencephalographic event-related potential (ERP) analyses and exact low-resolution brain electromagnetic tomography analyses (eLORETA) source localization analyses. Results showed that in cognitive and affective conditions, first-order trials elicited greater Anterior P2 (180–370 ms) amplitudes. In the cognitive condition, third-order trials elicited greatest amplitudes in the broadly distributed early negative slow wave (eNSW, 260–470 ms) and the late NSW (LNSW, 460–1,000 ms). In the affective condition, third-order and second-order trials elicited greatest amplitudes in a broadly distributed NSW (250–1,000 ms). Regarding affective trials in the NSW time span, statistical significant differences and trends were shown regarding activation of underlying brain regions. Third-order trials elicited greatest activation in a number of regions typically associated with the ToM network, especially the posterior cingulate cortex (PCC), cuneus, and temporoparietal junction (TPJ). Furthermore, ToM low performers (participants with high accuracy but longer reaction times) showed by trend smaller Posterior N1 and significantly smaller eNSW amplitudes compared to average and high performers. This study offers new insights into electrophysiological correlates of basic and higher order cognitive and affective ToM processing and its precise time course.

## Introduction

Theory of Mind (ToM) is defined as the attribution of mental states such as desires or intentions to oneself and others (e.g., Wellman, 2011). There are multiple methods to investigate specific aspects of ToM since there are multiple ways to predict another person's thoughts, motives, beliefs, and so on. In our social environment, very frequent cues for predicting another person's mental state are non-verbal cues like eye gaze, mimic, or body language (see e.g., Baron-Cohen et al., 1995; Frith and Frith, 1999). A look on one's watch or a gaze outside the window may indicate the urge to be somewhere else. If someone looks you in the eyes, you may observe said person's facial expression and conclude his or her feelings in the current situation. This essential tool of social interaction called ToM makes use of consecutive cognitive processing steps, each associated with activity in specific brain regions (see e.g., Abu-Akel and Shamay-Tsoory, 2011). Therefore, visual cues need to be perceived and interpreted, and the resulting data need to be processed so as to produce a meta-representation of the other person's mental state. In their neurobiological model, Abu-Akel and Shamay-Tsoory (2011) divide ToM processing into three basic steps that include (1) representing affective and cognitive mental states, (2) attributing these mental states to oneself or others, and (3) applying these mental states in order to understand and predict behavior. If multiple individuals are involved in a social interaction, it can be suggested that the previously mentioned series of cognitive processes needs to be expanded. This kind of expansion is described by the attribute “order” (see e.g., Perner and Wimmer, 1985). While the attribution of emotions, intentions, or beliefs to one individual is labeled “first-order ToM,” every additional step of attribution (e.g., “Harry thinks that Eva thinks …”) rises the order of ToM processing by one (e.g., Perner and Wimmer, 1985). Therefore, a social interaction in which one imagines what a person thinks about the thoughts of another person represents (second-order ToM) reasoning as the mental states of two individuals need to be considered.

The brain regions associated with ToM processing are already well-known and described in multiple reviews (e.g., Abu-Akel and Shamay-Tsoory, 2011; Poletti et al., 2012). ToM processing involves prefrontal regions such as the ventromedial prefrontal cortex (vmPFC) and the orbitofrontal cortex (OFC), both important in everyday preference judgments and processing emotions during decision making (see e.g., Bechara et al., 2000; Santos et al., 2011). Further prefrontal regions are the dorsolateral PFC (dlPFC) which is involved in higher order processes such as conscious decision making, reasoning, working memory, inhibition, and outcome prediction (see e.g., Krawczyk, 2002); the ventrolateral PFC (vlPFC) which is involved in behavior, speech, and reasoning execution (see e.g., Fuster, 2001); as well as the dorsomedial PFC (dmPFC) which is considered to hold a special role in representing other's mental states (see e.g., Lombardo et al., 2010). The importance of the dmPFC is further highlighted by a recent study by Bowman et al. (2019) which showed that this region is activated even in children as young as 4 years when performing first-order ToM tasks of various paradigms. Furthermore, multiple medial and posterior regions are involved such as the anterior cingulate cortex (ACC) which is, inter alia, associated with processing emotional aspects of self-reflection (see e.g., van der Meer et al., 2010), the posterior cingulate cortex (PCC) which is associated with processing self-mental states (see e.g., Lou et al., 2004), as well as the temporal pole (TP) seemingly activated in both cognitive and affective ToM (see e.g., Calabria et al., 2009; Lambon Ralph et al., 2009). Further regions are the precuneus which is associated with visuospatial imagery, episodic memory retrieval, and self-processing operations (Cavanna and Trimble, 2006); the cuneus which usually shows higher activity in cognitive ToM (Schlaffke et al., 2015); the temporoparietal junction (TPJ), not only associated with ToM processing but also with episodic memory, attention, and language processing (Igelström et al., 2015); as well as the superior temporal sulcus (STS), associated with the detection of social cues including prosody, faces, trustworthiness, and intention (Winston et al., 2002; Ethofer et al., 2006; Sabatinelli et al., 2011). Together, all these regions can be characterized as the neuronal ToM network (Poletti et al., 2012).

However, little is known regarding the chronological aspects of ToM-specific brain activity as, to date, most neuroscientific studies addressing ToM processing use either functional MRI (fMRI) which offers information about the activation of the previously mentioned brain regions but with a low temporal resolution or feature behavioral data of patients with brain lesions. Although recent fMRI studies show that multiple brain regions are activated during ToM processing (e.g., Xiao et al., 2018), the fMRI's temporal resolution allows only limited conclusions regarding the simultaneous or sequential activation of regions and therefore regarding the sequence of cognitive processes.

In order to investigate the chronological sequence of ToM processing and therefore build up a more profound knowledge regarding brain activity associated with ToM, studies featuring electroencephalographic event-related potentials (ERPs) are necessary. In this context, Liu et al. (2009) showed a ToM-related late slow wave (LSW) component at left frontal sites. This component was seen during first-order false belief reasoning tasks only in the recordings of those subjects who answered the presented false belief trials correctly. In a similar line of work, Zhang et al. (2006) showed a frontal late positive component (LPC), supposedly generated in the left middle frontal gyrus (based on LORETA analyses), associated with inhibiting one's own knowledge that may contradict the protagonist's knowledge. This indicates the importance of discriminating between the mental state of oneself and others (see e.g., Abu-Akel and Shamay-Tsoory, 2011). Meinhardt et al. (2011) designed an ERP task based on the “Sally and Anny” task by Baron-Cohen et al. (1985) in order to compare true and false belief reasoning in children and adults. The task consists of a series of pictures showing two characters in a room with an object and two places to put it. After the first character puts the object in one place (e.g., a box), the second character takes the object and puts it somewhere else. The first character either notices (in the true belief scenario) or does not notice (in the false belief scenario) this change. Their results revealed two ERP components that distinguished false belief reasoning from true belief reasoning: a late positive complex (LPC) that was most prominent at parietal sites and associated with the reorientation from external stimuli to internal mental representations, and an anterior LSW that was associated with the processing of internal mental representations regardless of external stimuli. Increases in the LSW amplitude in false belief trials compared to true belief trials were further associated with a higher amount of cognitive load inherent to false belief reasoning (Meinhardt et al., 2011). With respect to the LPC, Vistoli et al. (2015) conducted a study featuring sequential four-image comic strips presenting either intentional or physical contents. They found a bilateral posterior positive component (between 250 and 650 ms post-stimulus) which showed a greater amplitude in intentional than in physical conditions. In this way, they underpinned the time frame as well as the broad localization of intention processing as previously described by Meinhardt et al. (2011). Kühn-Popp et al. (2013) developed an ERP task similar to Meinhardt et al. (2011) that featured true belief, false belief, and pretense reasoning. Participants were shown common series of slides whereby each series was followed by a final slide that was specific for one of said three conditions. When comparing pretense reasoning to false belief reasoning, false belief elicited a positive frontocentral LSW between 290 and 920 post-stimulus (p.s.) which was associated with metarepresentation processing.

By and large, to date, there are few studies which shed light on cognitive processes involved in first-order ToM reasoning by featuring ERPs for precise temporal resolution. Furthermore, to the knowledge of the authors of the current study, there are none focusing on higher order ToM reasoning. Therefore, the overall aim of the current study was to present a task that contains first-, second-, and third-order trials of cognitive and affective ToM. Such a task would allow for the investigation of the electrophysiological correlates of basic as well as, for the first time, higher order ToM. For this reason, a ToM task based on visual cue processing was developed.

The developed task is based on the “Charlie Task” by Baron-Cohen et al. (1995) as well as on the “Yoni” task by Shamay-Tsoory and Aharon Peretz (2007). The “Charlie” task by Baron-Cohen et al. (1995) was created to study possible deficiencies in first-order ToM reasoning in autistic children compared to typically developing children and children with intellectual disabilities. The task focused on eye gazes which they defined as basic features in the course of grasping another person's intention. During the test, the examiner showed each child an A4 size slide with an image of a popular type of candy in each corner and a cartoon face in the middle that looked at one of these candies. While the typically developing children and the children with intellectual disabilities picked the correct answer when the subjects were asked what kind of candy “Charlie” wants, the autistic children were unable to guess the right answer and instead picked the candy they themselves liked best. Shamay-Tsoory and Aharon Peretz (2007) used a comparable method to prove their hypothesis that ToM processing depends on different regions of the frontal lobe as they investigated first- and second-order cognitive and affective ToM processing in patients with heterogeneous brain lesions. In the style of the Charlie task, a cartoon face (“Yoni”) was placed in the middle of a white screen with four objects belonging to the same category located in each corner. In the cognitive condition, “Yoni” gazed at one object, whereas in the affective condition, “Yoni” additionally smiled or frowned while a phrase was presented concerning Yoni's thoughts or feelings toward an object. In addition to these first-order tasks, second-order conditions were created by adding cartoon faces close to each object in the corner that also either just looked at one of the objects or additionally smiled or frowned. The results of this study led to the conclusion that an unimpaired PFC is necessary for successful cognitive and affective ToM. Affective ToM processing depends on the vmPFC, whereas cognitive ToM impairments are associated with lesions in the vmPFC and dlPFC (Shamay-Tsoory and Aharon Peretz, 2007). The Yoni task allowed for more elaborate investigations of ToM processing not just in healthy populations but also in schizophrenic or autistic patients as well as patients with Huntington's disease or dementia (see Poletti et al., 2012). Although yielding important information regarding cognitive and affective ToM processing in healthy and clinical populations, the scope of the original Yoni task is limited as it solely covers first- and second-order ToM reasoning and it only provides behavioral data.

Given the mentioned research on ToM processing, the aim of the current paper was therefore to investigate two aspects that were not or not sufficiently investigated previously, namely, the electrophysiological correlates of basic and higher-order ToM processing and its precise time course. Therefore, the current study presents an ERP task that includes not only first- but also second- and third-order tasks regarding both cognitive and affective ToM reasoning based on visual cues. This task is designed as a logical further development and ERP adaption of the validated, established, and well-documented “Charlie” and “Yoni” ToM tasks (Baron-Cohen et al., 1995; Shamay-Tsoory and Aharon Peretz, 2007). The current task allows for an investigation of the chronological sequence of basic and higher order ToM processing steps in a high temporal resolution. It further allows for an investigation of the electrophysiological brain correlates of different orders and types of ToM processing as well as for a low-resolution localization of respective activity using an electromagnetic tomography technique [exact low-resolution brain electromagnetic tomography analyses (eLORETA); see e.g., Pascual-Marqui et al., 2011]. This task therefore offers data that can be compared with previous behavioral and ERP studies on ToM processing. Furthermore, by combining the high temporal resolution of the electroencephalography (EEG) technique with validated (low-resolution) source localization analyses of the recorded data, the current study can to a certain degree indicate the activity of underlying brain regions during the early stages of ToM processing. This allows for complementary data to fMRI studies on ToM processing (which show high spatial resolution but low temporal resolution), whereas better results regarding the combination of temporal and spatial resolution could only be achieved by magnetoencephalography (MEG).

Another aim, building up on the exploration of the electrophysiological correlates of basic and higher order ToM processing, was to explore whether differences in electrophysiological activity can be found between ToM low and high performers (based on ToM performance in the form of accuracy and processing speed).

## Methods

### Sample

The study population included 20 right-handed [mean Edinburgh Handedness Inventory score = 0.85, *SD* = 0.14 (e.g., Oldfield, 1971)] students of the local medical university. The sample comprised individuals in emerging and early adulthood (20–33 years, *M* = 24.1 years, *SD* = 2.9 years) and a gender ratio of 50% females and 50% males. Participants were native German speakers of which all had a high school certificate and one additionally had a university degree. No participant reported a current or past neurological or psychiatric disease, and all participants had perfect or corrected to perfect vision. Before participation, all participants gave their written informed consent. The study protocol was approved by the institutional review board of the respective university and meets the ethical principles of the Declaration of Helsinki as well as the American Psychological Association (APA) ethical standards for human research.

### Stimuli and Task

*Brainy-ERP* (Deckert et al., 2016) is a computer task that was programmed with E-Prime 2.0 Professional (Psychology Software Tools^1^, Pittsburgh, PA). It is an electroencephalographic ERP adapted version of the “Brainy” task (Willinger et al., 2013) for the investigation of first-, second-, and third-order cognitive and affective ToM. This task is based on the established “Charlie” and “Yoni” ToM tasks (Baron-Cohen et al., 1995; Shamay-Tsoory and Aharon Peretz, 2007). The cognitive and affective first- and second-order trials were based on the “Yoni” task (Shamay-Tsoory and Aharon Peretz, 2007), whereas the third-order trials in both conditions were a logical further development based on the difference between the “Yoni” first- and second-order trials. In the following, the task will be described in detail.

*Brainy-ERP* consists of 510 trials, divided into two main conditions: cognitive (60 first-order, 70 second-order, and 80 third-order trials) and affective (80 first-order, 100 second-order, and 120 third-order trials). Each trial featured a sequence of slides with a brain-shaped cartoon face named “Brainy” in the middle of the screen and, in its proximity, one object belonging to one of nine different categories (cars, balls, leaves, fruits, chairs, animals, flowers, vegetables, and music instruments; 4–7 different object images per category). In order to reduce possible biases of physical properties of the stimuli in the EEG recordings, all objects included in this task were of equal size and on a gray scale (for possible biases, see e.g., Luck, 2014). In order to minimize eye movements during the task, the objects were placed in one of four predefined positions (top left, top right, bottom left, and bottom right) close to Brainy which were chosen in a pseudorandomized fashion and equal distribution.

**Figure 1:**
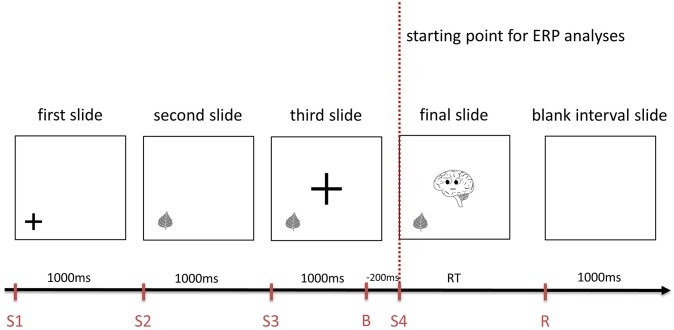
An example of the sequence of slides composing a cognitive first order Theory of Mind trial of *Brainy-ERP*. Each slide is shown for 1,000 ms before the next one appears. The final slide is shown until the response is given, followed by a 1,000 ms interval until the first slide of the next trial is shown (S1-4, starting point of each slide; B, baseline for ERP analyses, R, response; RT, response time).

**Figure 2:**
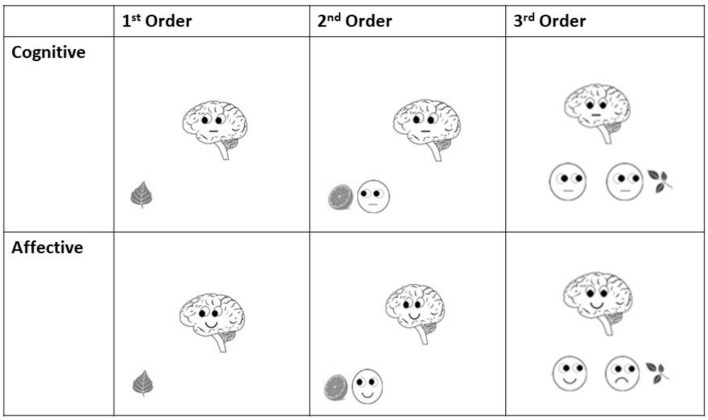
Examples of final slides (starting point for ERP analyses) of each order in both cognitive and affective Theory of Mind trials of *Brainy-ERP*.

Prior to those sequences, a statement was presented. The consecutive slides gradually gave the subject the information needed for the decision whether the previous statement is true or false (Figure 1), whereas Brainy as the final cue was provided on the last slide. This final slide served as the starting point for the ERP analyses in this study (Figures 1, 2). The participants had to decide whether the statement fitted the final slide of the sequence and had to push a “YES” or a “NO” button (n and m, respectively, of a QWERTZU keyboard) with their right index and middle fingers. These statements were, for example, “*Brainy thinks of this object*” (cognitive ToM, first order), “*Brainy likes this object*” (affective ToM, first order), “*Brainy thinks that someone else thinks about that object*” (cognitive ToM, second order), “*Brainy dislikes that someone else likes this object*” (affective ToM, second order), “*Brainy thinks that someone else thinks that another person thinks about that object*” (cognitive ToM, third order), and “*Brainy likes that someone else dislikes that another person likes this object*” (affective ToM, third order). The other persons whose mental states had to be taken into account were represented by smileys which were only featured in second- and third-order trials. With respect to affective ToM trial statements, the combinations of “likes/dislikes” in the statements regarding the second and third orders were chosen in a pseudorandomized fashion and equal distribution.

Similar to adding protagonists in false belief tasks (see e.g., Valle et al., 2015) as well as in the style of Shamay-Tsoory and Aharon-Peretz (2010), these additional smileys were included in order to induce additional steps of recursive thinking needed for higher order ToM processing. In first-order trials (Figure 1), participants had only to take Brainy's eye gaze (cognitive trials) or Brainy's eye gaze and mimic into account (affective trials), whereas in second-order ToM trials, the participants additionally had to take the visual cues of a smiley into account which appeared next to the object before Brainy appeared. In the third-order trials, after the slide with the smiley next to the object was shown, on the following slide, a second smiley appeared close to the first one. As the final slide (Brainy) was presented, the participants needed to consider both smileys' as well as Brainy's eye gaze and mimic so as to process the prior shown statement. In the cognitive condition, Brainy and the smileys showed a neutral expression, whereas in the affective condition, Brainy and the smileys either smiled or showed an unhappy expression (Figure 2).

In the form of a *stage processing ToM paradigm*, all slides were presented one after another (Figure 1), consecutively adding information. The order of informative slides was “object—Brainy” for first-order trials, “object—smiley—Brainy” for second-order trials, and “object—smiley 1—smiley 2—Brainy” for third-order trials. Before each informative slide, cuing slides were inserted on which a fixation cross showed the spot where an object, smiley, or Brainy appeared in the following slide, reducing eye movement-induced EEG artifacts. The fixation crosses had two standard sizes, one smaller fitting the objects as well as the smileys, and one bigger fitting Brainy. After the participant's response, the next trial started after a 1000 ms blank slide interval to avoid overlapping ERP components elicited by the response or the visual input of the first slide of the next trial (for an overview, see Figure 1).

Participants had to decide whether a statement (e.g., “*Brainy dislikes that someone else likes this object*”) matched the following slides. In this way, Brainy's or the smiley's eye gazes or expressions either matched or did not match the information given in the statement allowing for “right trials” (all information match—participants ideally answer with “Yes”) or “false trials” (one or more pieces of information of slides and statement do not match—participants ideally answer with “No”). Within false trials, not matching eye gazes or expressions could either be shown by Brainy, the smiley, or both. Not matching information was shown at different positions in slide sequence (e.g., shown on the final slide by Brainy; shown by smiley 2 before the final slide; …) in order to ensure that participants focused on each ToM processing step (each slide) throughout the task. However, those trials in which a smiley's eye gaze or expression did not match the previously shown statement were excluded from the ERP analysis of the present paper. In these trials, ToM processing is most likely completed and the decision is made prior to the final slide. In such cases, participants would have already known before the final slide that the specific trial was incorrect, hence finishing the cognitive process before the starting point of the ERP recording which would lead to biases in ERP data.

The cognitive part of the Brainy task consisted of 40 right and 20 false trials for first order; 40 right, 20 false (Brainy), and 10 false (Smiley) for second order; and 40 right, 20 false (Brainy), 10 false (Smiley 1), and 10 false (Smiley 2) trials for third order. A false trial of cognitive ToM always included a wrong direction of the eye gaze.

The affective part of the Brainy task consisted of 40 right and 40 false trials for first order; 40 right, 40 false (Brainy), and 20 false (Smiley) for second order; and 40 right, 40 false (Brainy), 20 false (Smiley 1), and 20 false (Smiley 2) trials for third order. The false trials of affective ToM always included either a wrong direction of the eye gaze or wrong expression (of Brainy or the Smiley, respectively). Error types (eye gaze or expression errors) were equally distributed.

### Procedure

The study took place in an electrically shielded, sound-attenuated chamber in which the participants sat comfortably in a padded chair, 27 inches (70 cm) away from a 19-inch computer monitor at which they looked in an average visual angle of 4.9°. While the EEG was recorded, subjects started with the cognitive part of the *Brainy-ERP* task, followed by an obligatory 15-min break before continuing with the affective part. Test duration was approximately 65 min (25 min for 210 cognitive trials and 40 min for 300 affective trials; average duration of 7.64 s per trial). The order of the parts was chosen so as to start with a smaller number of visual cues (only eye gazes) and to increase them afterward (eye gazes and expressions). Additionally, participants filled out a sociodemographic form as well as the Edinburgh Handedness Inventory (Oldfield, 1971) while being prepared for the EEG recording.

### Electroencephalography—Event-Related Potentials

Electrophysiological activity of the brain was recorded with a 64-channel amplifier (BrainAmp-Standard, BrainProducts GmbH) and 64-channel Easycap electrode caps using sintered Ag/AgCl electrodes (Easycap GmbH). The electrodes had fixed positions in accordance with the extended International 10–20 system. The reference electrode was FCz, whereas the ground electrode was AFz. FCz was chosen as the reference electrode as it avoids a hemisphere bias and, as a central electrode, it is expected to interfere minimally with underlying brain regions of interest [see brain regions associated with ToM processing such as prefrontal, temporal (speaking against linked mastoids reference), and parietal regions] as well as visual regions. For the absence of “neutral” sites as well as the disadvantages of average referentiation, see Luck (2014). For the detection of eye movements, two horizontal ocular electrodes were positioned at the outer canthus of each eye and a vertical ocular electrode below the left eye, leaving 61 scalp electrodes. The activity was filtered online with a bandpass filter (0.016–200 Hz), and the sampling rate was 1,000 Hz. Raw data were then analyzed with the Brain Vision Analyzer Version 2.0.2 (BrainProducts GmbH). After filtering raw data with a IIR bandpass filter (0.1–40 Hz), artifacts (horizontal and vertical eye movements and others) were removed in the course of a semiautomatic raw-data inspection (*gradient*: maximal allowed voltage step of 50 μV/between two data points; *max-min*: maximal allowed absolute difference of 200 μV within an interval length of 200; *low activity*: lowest allowed activity of 0.5 μV within an interval length of 100) as well as a manual artifact rejection (visual inspection) based on the guidelines of Luck (2014). The final slide of each trial (Figure 1) was the starting point for the ERP analyses, whereby the baseline correction period was set to −200 ms before the final slide and activity between 0 and 1,000 p.s. was analyzed.

After building grand averages of aforementioned segments regarding first-, second-, and third-order cognitive and affective trials separately, these grand average segments were overlaid. On basis of these overlays, all 61 active electrodes were inspected, and ERP components were visually identified. For each of the identified components, those electrodes were pooled for which similar time spans and voltage amplitudes could be identified and which showed spatial proximity (electrodes next to another). As the aim of the paper was to find differences in electrophysiological activity regarding processing different orders of cognitive and affective ToM trials, only those components were chosen for statistical analyses for which differences between at least two orders could initially be visually identified. With respect to classic visual ERP components such as P1, anterior N1, and posterior N1 (for an overview, see e.g., Luck and Kappenman, 2011), no meaningful differences between orders were identified, whereas for following components, differences were identified upon visual inspection.

#### Anterior P2

In the time span of 180–370 ms after presentation of the final slide (p.s.), differences between orders were identified regarding the P2 component at anterior sites in both cognitive and affective trials. Whereas, P2 can be seen as a visual component, it nevertheless depends on cognitive factors such as identifying target features of stimuli (for an overview, see e.g., Luck, 2014). Please note that the final slide always presents Brainy whereas its eye gazes and expressions only vary across items (depending on the statement) but apart from that always looks the same across orders. Slightly different sites of P2 were shown bilaterally for cognitive (Fp1, Fp2, AF3, AF4, AF7, AF8, F3, F4, F5, F6, F7, F8, FC3, FC4, FC5, FC6, FT7, FT8, C3, C4, C5, C6, T7, T8) and affective trials (Fp1, Fp2, AF3, AF4, AF7, AF8, F3, F4, F5, F6, F7, F8, FC3, FC4, FC5, FC6, FT7, FT8, C5, C6, T7, T8).

#### Affective Negative Slow Wave

In the time span of 250–1,000 ms p.s., differences between orders in affective trials were seen regarding a broadly distributed slow wave [negative slow wave (NSW)]. This component is comparable to a slow wave shown in Kühn-Popp et al. (2013). The NSW was seen at a number of broadly distributed bilateral sites: AF3, AF7, F3, F5, F6, F7, F8, FC3, FC5, FC6, FT7, FT8, C3, C5, C6, T7, T8 CP3, CP5, CP6, TP7, TP8, TP9, TP10, P3, P5, P6, P7, P8.

#### Cognitive Early Negative Slow Wave

In the time span of 350–470 ms p.s., differences between orders could be identified regarding a broadly distributed negative component which was named early negative slow wave (eNSW). Whereas, the time span of the component is similar to the LPC component by Zhang et al. (2006), it is a negative deflection with a broad bilateral distribution (AF3, AF7, F3, F5, F6, F7, F8, FC3, FC5, FT7, C3, C5, C6, T7, T8, CP3, CP5, CP6, TP7, TP9, P3, P5, P6, P7, P8).

#### Cognitive Late Negative Slow Wave

In the time span of 460–1,000 ms p.s., differences between orders were shown regarding a late NSW (LNSW) which also was broadly bilaterally distributed (Fp1, Fp2, AF3, AF4, AF7, AF8, F2, F3, F4, F5, F6, F7, F8, FC3, FC5, FT7, C5, T7, CP5, TP7, TP9, P5, P7, PO7). Interestingly, the eNSW and the LNSW components together spread the time span of the affective NSW, and therefore together resemble the time aspects of the slow wave shown in Kühn-Popp et al. (2013).

As another aim of the study was to identify electrophysiological differences between ToM high and low performers (for definition of the groups, see the statistics part), the EEG data were again similarly analyzed whether visual differences in grand average voltage amplitudes can be identified. Besides the previously mentioned ERP components, visual differences between performer groups could be identified in the posterior N1 component in the time span of 120–250 ms p.s. at parieto-occipital sites (Pz, P1, P2, P3, P4, P5, P6, P7, P8, POz, PO3, PO4, PO7, PO8, Oz, O1, O2) for both cognitive and affective trials. Greater amplitudes in the N1 component were previously associated with discrimination tasks (for an overview, see e.g., Luck and Kappenman, 2011).

### Source Localization

Regarding those components for which significant differences between orders were shown, eLORETA (see e.g., Pascual-Marqui, 2007, 2009; Pascual-Marqui et al., 2011) were performed for the time span of the respective component. eLORETA have been already validated and the software as well as the validation studies are freely available at http://www.uzh.ch/keyinst/loreta.htm. Based on the scalp-recorded electric potential distribution (frontopolar, anterior frontal, frontal, frontocentral, frontotemporal, central, centroparietal, temporal, temporoparietal, parietal, parietooccipital, and occipital sites), this program estimates the origins of neural currents (for approaches to solve the “inverse problem” in EEG recordings, see e.g., Luck, 2014) in order to provide an approximation of the cortical three-dimensional (3D) distribution of electrophysiological brain activity in terms of a current density field. eLORETA is based on a discrete, 3D distributed, linear, weighted minimum norm inverse solution that computes current density with exact localization but low spatial resolution. It provides the estimated localization of Brodmann areas (BAs) as well as predefined regions of interest (ROIs) within a source space of 6,239 voxels at 5-mm spatial resolution that covers cortical gray matter and the hippocampus. Whereas other localization techniques are strongly influenced by basic principles of electricity such as volume conduction (i.e., electricity spreading across a conductive medium instead of directly running between two poles of dipole when located in a conductor, see Luck, 2014), eLORETA use, inter alia, lagged components which were shown to have an almost “pure” physiological origin (Pascual-Marqui et al., 2011). Validation of localization properties of eLORETA was, inter alia, obtained from source localization of primary and secondary sensory cortices upon usage of visual, auditory, and somatosensory ERPs (see e.g., Pascual-Marqui et al., 2011). The mean current density field in the respective areas (μA/mm^2^) can be exported and statistically compared. Calculations are made in a realistic head model (Fuchs et al., 2002) using the MNI-152 template (Mazziotta et al., 2001) including the standard electrode positions on the MNI-152 scalp (Oostenveld and Praamstra, 2001; Jurcak et al., 2007) as well as the 3D solution space determined by the Talairach atlas (Lancaster et al., 2000). For visualization, eLORETA images show the electric activity at each voxel in the neuroanatomic Montreal Neurological Institute (MNI, McGill University) space (involving MNI coordinates *x, y, z*) as the precise magnitude of the estimated current density.

Based on literature on ToM-specific activation of brain regions, the following ROIs were chosen for statistical comparisons: bilateral dlPFC (BAs 9, 46), bilateral dmPFC (BA 8), bilateral vlPFC (BAs 44, 45, 47), bilateral vmPFC (BAs 10, 11), ACC (predefined region by eLORETA, including BAs 24, 25, 32), PCC (predefined region, including BAs 18, 23, 29, 30, 31), bilateral cuneus (predefined region, including BAs 7, 17, 18, 19, as well as parts of BAs 23, 30, 31), bilateral precuneus (predefined region, including BAs 7, 18, 19, 23, 31, 39), bilateral TP (BA 38), bilateral STS (BAs 21, 22), and the bilateral TPJ (BAs 39, 40). BAs for prefrontal regions were defined based on Carlén (2017), whereas BA 8 was assigned to the dmPFC and BA 9 to the dlPFC. The remaining BAs were either based on Abu-Akel and Shamay-Tsoory (2011) or given by eLORETA program.

### Statistical Analyses

Differences regarding behavioral variables between conditions and orders were analyzed for significance with repeated-measures analyses of variance (rmANOVAs) including response accuracy percentage as well as response time as dependent variables and the factors “condition” (affective, cognitive) and “order” (first, second, and third) as independent variables.

In order to analyze relations between cognitive and affective ToM with respect to behavioral variables, Pearson correlations between response times as well as between response accuracy percentages during cognitive and affective trials were conducted for each order separately.

In order to compare different performer groups regarding ToM performance, the sample was divided into the fastest 25% (“fast responder,” *n* = 5), middle 50% (“average responder,” *n* = 10), and the slowest 25% (“slow responder,” *n* = 5) based on the total response time. No performance group was built on total accuracy percentage as no significant differences between orders or conditions as well as no interaction effect were shown (see the Results section). Furthermore, regarding accuracy percentage, a ceiling effect can be seen (Table 1). Differences in response accuracy percentage between performer groups were analyzed using rmANOVAs for each order in both conditions.

**Table 1:** Descriptive data regarding *Brainy-ERP*.

**Task**	**Score range**	**Min. score (%)**	**Max. score (%)**	**Mean score (*SD*)**	**Mean accuracy % (*SD*)**	**Mean RT in milliseconds (*SD*)**
**Cognitive ToM**						
First order	0–60	34 (57%)	60 (100%)	55.1 (7.27)	91.83 (12.11)	1114.4 (622)
Second order	0–70	33 (47%)	70 (100%)	64.95 (10.43)	92.79 (14.90)	938.8 (694.9)
Third order	0–80	44 (55%)	80 (100%)	76.25 (8.89)	95.31 (11.11)	948.4 (707.1)
TOTAL score	0–210	137 (65%)	210 (100%)	196.30 (23.65)	93.48 (11.26)	1000.5 (646.2)
**Affective ToM**						
First order	0–80	58 (73%)	80 (100%)	75.65 (5.39)	94.56 (6.74)	1091.9 (622.3)
Second order	0–100	65 (65%)	99 (99%)	93.35 (8.77)	93.35 (8.77)	917.7 (346.2)
Third order	0–120	83 (69%)	118 (98%)	112.10 (8.25)	93.42 (6.87)	938.3 (286.2)
TOTAL score	0–300	214 (71%)	296 (99%)	281.10 (20.57)	93.70 (6.86)	982.6 (396.4)

*ToM, Theory of Mind*.

Differences in mean amplitudes regarding previously described ERP components were analyzed for significance using rmANOVAs with the factor “order” (first, second, and third) as well as additionally the factor “hemisphere” (right vs. left) if components were bilaterally distributed. rmANOVAs were performed for both conditions separately.

In order to analyze relations between cognitive and affective ToM with respect to ERP components, Pearson correlations between respective mean amplitudes regarding cognitive and affective trials were conducted for each order separately.

Differences in mean amplitude regarding previously described ERP components between performer groups were analyzed using rmANOVAs for each order in both conditions.

Current density in the respective ROIs was calculated in the time spans in which significant differences between orders regarding ERP components were found (e.g., 250–1,000 ms in affective trials, see the affective NSW). Differences in current density means were analyzed for significance using rmANOVAs with the factor “order” (first, second, and third) as well as additionally the factor “hemisphere” (right vs. left) if ROIs were bilaterally distributed. rmANOVAs were performed for both conditions separately.

The *p*-value for statistical significance was set at *p* < 0.05. If necessary, *p*-values were adjusted using the Greenhouse–Geisser correction. *Post-hoc* tests (Bonferroni) were applied if main or interaction effects were significant. In case of multiple comparisons, the Bonferroni–Holm method was implemented to prevent type I errors, and the corrected *p*-value thresholds were marked with an asterisk (e.g., “^*^p”).

## Results

### Behavioral Results

The behavioral results regarding the different orders and conditions of Brainy-ERP are listed in Table 1. Mean response accuracy was high in each subset of Brainy-ERP and shows a slight increase from the first to the third order in the cognitive condition and similar values in the first to the third order in the affective condition. In contrast, the response time of the participants in each condition was longest in the first order and shortest in the second order (Table 1).

rmANOVA regarding response accuracy percentage revealed no significant differences regarding either condition, *F*_(1, 19)_ = 0.08, *p* = 0.78, order, *F*_(1, 19)_ = 0.53, *p* = 0.59, or the interaction of both, *F*_(1, 19)_ = 1.83, *p* = 0.18. Therefore, it can be concluded that all parts of the task were understood, enabling a successful ToM processing recording (Table 1).

rmANOVA regarding response time showed a highly significant main effect of order, *F*_(2, 38)_ = 12.13, *p* ≤ 0.0001, but not regarding condition, *F*_(1, 19)_ = 1.36, *p* = 0.26, as well as no interaction effect of order and condition, *F*_(1.24,23.56)_ = 0.22, *p* = 0.7. Inner subject contrasts revealed highly significantly faster mean response times in third-order than in first-order trials, *F*_(1, 19)_ = 15.76, *p* = 0.00082, as well as in second-order compared to first-order trials, *F*_(1, 19)_ = 22.43, *p* = 0.00014. However, no significant differences between third- and second-order trials were shown, *F*_(1, 19)_ = 0.99, *p* = 0.33 (Table 1).

rmANOVAs showed no significant differences in response accuracy percentage between (response time) performer groups regarding cognitive first order, *F*_(2, 17)_ = 0.666, *p* = 0.527, cognitive second order, *F*_(2, 17)_ = 0.841, *p* = 0.448, cognitive third order, *F*_(2, 17)_ = 5.035, *p* = 0.019 (^*^p = 0.016), affective first order, *F*_(2, 17)_ = 1.527, *p* = 0.246, affective second order, *F*_(2, 17)_ = 3.454, *p* = 0.055, as well as affective third order, *F*_(2, 17)_ = 4.039, *p* = 0.0347 (^*^p = 0.016).

Pearson correlations between response times as well as between response accuracy percentages during cognitive and affective trials revealed medium to high correlations (Table 2).

**Table 2:** Pearson correlations between response times of cognitive and affective ToM trials for each order as well as between the mean amplitudes of the found ERP components.

**Behavioral results**										
**Response times**							**Response accuracy percentage**			
	**Cognitive trials**							**Cognitive trials**		
	**1. order**	**2. order**	**3. order**					**1. order**	**2. order**	**3. order**
**Affective trials**							**Affective trials**			
1. order	0.874^***^	0.956^***^	0.916^***^				1. order	0.770^***^	0.778^***^	0.642^**^
2. order	0.887^***^	0.929^***^	0.885^***^				2. order	0.429	0.544^*^	0.944^***^
3. order	0.776^***^	0.689^**^	0.811^***^				3. order	0.483^*^	0.472^*^	0.733^***^
**ERP components**										
	**Cognitive aP2 (180–370 ms)**									
	**1. order**	**2. order**	**3. order**							
**Affective aP2 (180–370 ms)**										
1. order	0.828^***^	0.552^*^	0.766^***^							
2. order	0.584^**^	0.500^*^	0.848^***^							
3. order	0.456^*^	0.455^*^	0.890^***^							
	**Cognitive eNSW (260–470 ms)**			**Cognitive LNSW (460–1,000 ms)**				**^†^Combined cognitive NSW (260–1,000 ms)**		
	**1. order**	**2. order**	**3. order**	**1. order**	**2. order**	**3. order**		**1. order**	**2. order**	**3. order**
**Affective NSW (250–1,000 ms)**										
1. order	0.548^*^	0.475^*^	0.523^*^	0.777^***^	0.803^***^	0.619^**^		0.879^***^	0.729^***^	0.652^**^
2. order	0.638^**^	0.577^**^	0.860^***^	0.455^*^	0.707^***^	0.671^**^		0.618^**^	0.585^**^	0.737^***^
3. order	0.546^*^	0.531^*^	0.667^**^	0.571^**^	0.755^***^	0.717^***^		0.674^**^	0.640^**^	0.770^***^

*ToM, Theory of Mind; ERP, Electroencephalographic Event-related potentials*.

*ERP components: aP2, anterior P2; eNSW, early negative slow wave; LNSW, late negative slow wave; NSW, negative slow wave*.

† *Besides comparing the negative slow waves between cognitive and affective trials as they were shown in the study, an additional combined cognitive negative slow wave was calculated*.

*This combined NSW is the mean of the eNSW and LNSW and was calculated in order to compare slow waves of similar length (affective: 250–1,000 ms, cognitive: 260–1,000 ms)*.

*** *≤ 0.001*;

** ≤ 0.01; and

* *≤ 0.05*.

### Electroencephalographic Event-Related Potential Components—Cognitive Theory of Mind

#### Anterior P2 (180–370 ms Post-stimulus)

**Figure 3:**
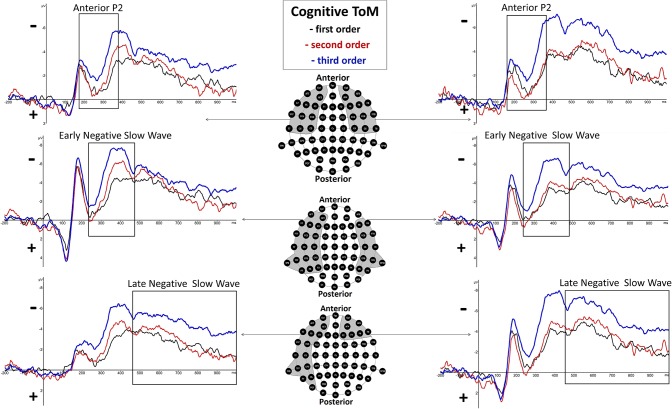
Significant differences between Theory of Mind (ToM) first order (black line), second order (red line), and third order (blue line) trials were shown regarding Anterior P2 (180–370 ms), Early Negative Slow Wave (ENSW, 260–470 ms), and Late Negative Slow Wave (LNSW, 460–1,000 ms) mean amplitudes at respective pooled sites (gray areas). Graphs represent the EEG-ERP data that was statistically analyzed (see “Electroencephalographic Event-Related Potential Components—Cognitive Theory of Mind” in the result section).

There was a highly significant main effect of order shown, *F*_(2, 38)_ = 11.28, *p* = 0.000143 (^*^p = 0.025), but none regarding hemisphere, *F*_(1, 19)_ = 0.02, *p* = 0.89, as well as no significant interaction effect of order and hemisphere, *F*_(2, 38)_ = 1.98, *p* = 0.15. Inner subject contrasts revealed a highly significantly more positive mean amplitude of second-order trials (*M* = −1.5 μV, *SD* = 2.191) compared to third-order trials (*M* = −3.242 μV, *SD* = 1.934), *F*_(1, 19)_ = 19.27, *p* = 0.000315, as well as a significantly more positive mean amplitude of first-order trials (*M* = −1.525 μV, *SD* = 1.968) compared to third-order trials, *F*_(1, 19)_ = 18.63, *p* = 0.000372. However, inner subject contrasts showed no difference regarding mean amplitude of the second order compared to the first order, *F*_(1, 19)_ = 0.003, *p* = 0.958. For a graphical representation, see Figure 3.

#### Early Negative Slow Wave (260–470 ms Post-stimulus)

There was a highly significant main effect of order shown, *F*_(2, 38)_ = 13.17, *p* = 0.000045 (^*^p = 0.0166), but none regarding hemisphere, *F*_(1, 19)_ = 4.81, *p* = 0.04, as well as no significant interaction effect of order and hemisphere, *F*_(2, 38)_ = 2.1, *p* = 0.14. Inner subject contrasts revealed a significantly more negative mean amplitude of the third order (*M* = −5.312 μV, *SD* = 3.043) than the second order (*M* = −3.262 μV, *SD* = 3.205), *F*_(1, 19)_ = 11.61, *p* = 0.003, and highly significantly more than the first order (*M* = −2.637 μV, *SD* = 2.734), *F*_(1, 19)_ = 28.13, *p* = 0.000041. However, inner subject contrasts showed no difference regarding mean amplitude of the second order compared to the first order, *F*_(1, 19)_ = 1.811, *p* = 0.194. For a graphical representation, see Figure 3.

#### Late Negative Slow Wave (460–1,000 ms Post-stimulus)

There was a highly significant main effect of order shown, *F*_(1.5,28.4)_ = 12.51, *p* = 0.000388 (^*^p = 0.05), and a significant main effect of hemisphere, *F*_(1, 19)_ = 5.59, *p* = 0.03, but none regarding interaction effect of order and hemisphere, *F*_(2, 38)_ = 0.21, *p* = 0.81, with inner subject contrasts revealing a highly significantly more negative mean amplitude of the third order (*M* = −4.905 μV, *SD* = 3.392) than the second order (*M* = −3.043 μV, *SD* = 2.101), *F*_(1, 19)_ = 19.585, *p* = 0.00029, and highly significantly more than the first order (*M* = −2.781 μV, *SD* = 2.833), *F*_(1, 19)_ = 13.17, *p* = 0.00165. However, inner subject contrasts showed no difference regarding mean amplitude of the second order compared to the first order, *F*_(1, 19)_ = 0.527, *p* = 0.477. For a graphical representation, see Figure 3. The topographical distributions of voltages across the whole scalp for the time spans of the respective ERP components are shown in **Figure 5**.

### Electroencephalographic Event-Related Potential Components—Affective Theory of Mind

#### Anterior P2 (180–370 ms Post-stimulus)

**Figure 4:**
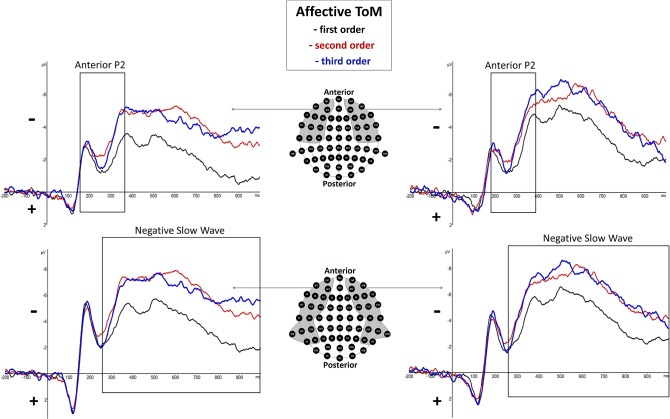
Significant differences between Theory of Mind (ToM) first order (black line), second order (red line), and third order (blue line) trials were shown regarding Anterior P2 (180–370 ms) and Negative Slow Wave (NSW, 250–1,000 ms) mean amplitudes at respective pooled sites (gray areas). Graphs represent the EEG-ERP data that was statistically analyzed (see “Electroencephalographic Event-Related Potential Components—Affective Theory of Mind” in the results section).

There was a significant main effect of order shown, *F*_(2, 38)_ = 5.53, *p* = 0.008 (^*^p = 0.05), but none regarding hemisphere, *F*_(1, 19)_ = 0.29, *p* = 0.6, as well as no significant interaction effect of order and hemisphere, *F*_(2, 38)_ = 1.38, *p* = 0.27. However, inner subject contrasts showed a significantly more positive mean amplitude of first-order trials (*M* = −2.241 μV, *SD* = 2.008) compared to third-order trials (*M* = −3.139 μV, *SD* = 2.143), *F*_(1, 19)_ = 7.09, *p* = 0.015, and second-order trials (*M* = −3.207 μV, *SD* = 2.427), *F*_(1, 19)_ = 8.77, *p* = 0.008. However, inner subject contrasts showed no difference between third-order and second-order trials, *F*_(1, 19)_ = 0.049, *p* = 0.828. For a graphical representation, see Figure 4.

#### Negative Slow Wave (250–1,000 ms Post-stimulus)

**Figure 5:**
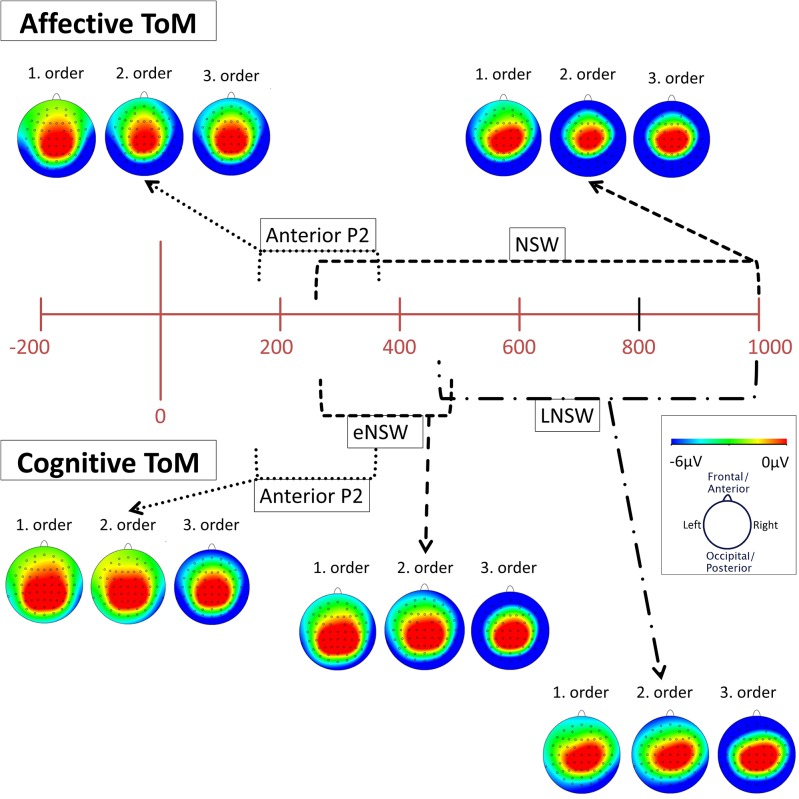
Topographical distribution (mapping view) of mean voltage in the time spans of the identified event-related potential (ERP) components: cognitive and affective Anterior P2 (180–370 ms), affective negative slow wave (NSW, 250–1,000 ms), cognitive early negative slow wave (eNSW, 260–470 ms), and cognitive late negative slow wave (LNSW, 460–1,000 ms). The voltage distribution is shown for each order in the respective time span. The small black dots across each head represent the 61 active scalp electrodes. Note that the voltages become more negative at frontal and lateral sites across orders.

There was a highly significant main effect of order shown, *F*_(2, 38)_ = 15.14, *p* = 0.000015 (^*^p = 0.025), but no significant main effect of hemisphere, *F*_(1, 19)_ = 0.11, *p* = 0.75, as well as no significant interaction effect of order and hemisphere, *F*_(2, 38)_ = 4.13, *p* = 0.04. Inner subject contrasts revealed a highly significantly more negative mean amplitude of the third order (*M* = −6.107 μV, *SD* = 2.745) than the first order (*M* = −3.949 μV, *SD* = 2.253), *F*_(1, 19)_ = 30.48, *p* = 0.000025, as well as a significantly more negative mean amplitude of the second order (*M* = −6.053 μV, *SD* = 2.937) compared to the first order, *F*_(1, 19)_ = 18.92, *p* = 0.000345. However, inner subject contrasts showed no difference regarding mean amplitude of the third order compared to the second order, *F*_(1, 19)_ = 0.014, *p* = 0.908. For a graphical representation, see Figure 4. The topographical distributions of voltages across the whole scalp for the time spans of the respective ERP components are shown in Figure 5.

### Electroencephalographic Event-Related Potential Components—Relation Between Cognitive and Affective Theory of Mind

Pearson correlations between mean amplitudes of cognitive and affective ERP components revealed medium to high correlations (Table 2).

### Electroencephalographic Event-Related Potential Components—Theory of Mind High Performer vs. Theory of Mind Low Performer

**Figure 6:**
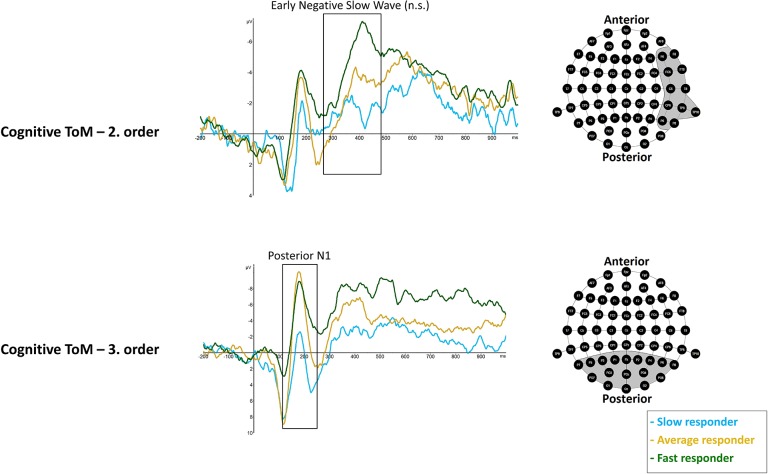
Significant differences between slow (light blue line), average (dark yellow), and fast responder (dark green line) were shown in cognitive ToM third order's Posterior N1 (120–250 ms) mean amplitude at respective pooled sites (gray area). Regarding cognitive ToM second order's Early Negative Slow Wave (260–470 ms) a statistical trend was shown. Please note that the sequence of ToM trials across the whole task was: cognitive ToM first order, second order, and then third order as well as afterwards affective ToM first, second, and then third order. Graphs represent the EEG-ERP data that was statistically analyzed (see “Electroencephalographic Event-Related Potential Components—Theory of Mind High Performer vs. Theory of Mind Low Performer” in the results section).

rmMANOVAs showed significant differences between performer groups with respect to cognitive third-order posterior N1 mean amplitude, *F*_(2, 17)_ = 6.023, *p* = 0.0105 (^*^p = 0.016). *Post hoc* analyses showed significantly more negative posterior N1 mean amplitudes in fast responders (*M* = −4.194 μV, *SD* = 1.414) compared to slow responders (*M* = 2.455 μV, *SD* = 1.414), *p* = 0.012, as well as more negative mean amplitudes in average responders (*M* = −2.274 μV, *SD* = 1.0), compared to slow responders, *p* = 0.043. No differences were revealed between fast and average responders, *p* = 0.849 (Figure 6).

rmMANOVAs showed a statistical trend with respect to differences between performer groups in right-hemisphere ENSW in second-order cognitive trials, *F*_(2, 17)_ = 4.03, *p* = 0.037 (^*^p = 0.025). Results indicate more negative right-hemisphere ENSW amplitudes in fast responders (*M* = −7.081 μV, *SD* = 2.371) compared to slow responders (*M* = −1.591 μV, *SD* = 1.444), *p* = 0.035, but no differences were indicated between fast and average responders (*M* = −4.028 μV, *SD* = 3.788), *p* = 0.261 as well as between average responders and slow responders, *p* = 0.495 (Figure 6).

### Source Localization [Exact Low-Resolution Brain Electromagnetic Tomography Analyses (eLORETA)]—Cognitive Theory of Mind

In the following, only significant results as well as statistical trends will be presented. The complete list of results can be found in the supplemental.

#### Time Span of the Early Negative Slow Wave Component (350–470 ms Post-stimulus)

Regarding the PCC, there was a statistical trend regarding the main effect of order, *F*_(2, 38)_ = 5.115, *p* = 0.011 (^*^p = 0.004). Inner subject contrasts indicated stronger activation in third-order (*M* = 0.03 μA/mm^2^, *SD* = 0.021) compared to first-order trials (*M* = 0.022 μA/mm^2^, *SD* = 0.015), *F*_(1, 19)_ = 5.519, *p* = 0.030, and second-order trials (*M* = 0.022 μA/mm^2^, *SD* = 0.014), *F*_(1, 19)_ = 7.51, *p* = 0.013. No differences were indicated between second-order and first-order trials, *F*_(1, 19)_ = 0.009, *p* = 0.927.

#### Time Span of the Late Negative Slow Wave Component (460–1,000 ms Post-stimulus)

Regarding the PCC, no significant main effect of order was shown, *F*_(1.551, 29.467)_ = 3.554, *p* = 0.0519. Nevertheless, inner subject contrasts indicated stronger activation in third-order (*M* = 0.016 μA/mm^2^, *SD* = 0.012) compared to first-order trials (*M* = 0.012 μA/mm^2^, *SD* = 0.008), *F*_(1, 19)_ = 5.086, *p* = 0.0361.

### Source Localization [Exact Low-Resolution Brain Electromagnetic Tomography Analyses (eLORETA)]—Affective Theory of Mind

#### Time Span of the Negative Slow Wave Component (250–1,000 ms Post-stimulus)

**Figure 7:**
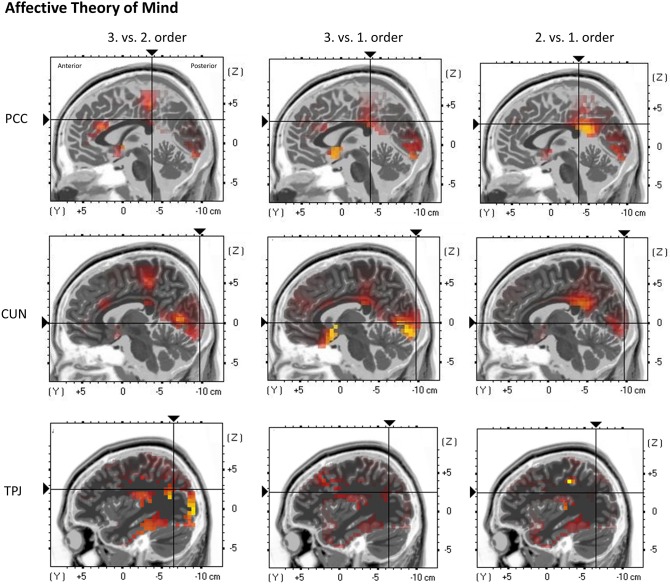
eLORETA source localization shows differences in electrophysiological activity (current density, μA/mm^2^) between the three ToM orders in the Posterior Cingulate Cortex (PCC—top panel), the Cuneus (CUN—middle panel), and the Temporoparietal Junction (TPJ—bottom panel) in affective ToM trials within the time span of the Negative Slow Wave (NSW, 250–1,000 ms). The third order elicits the greatest activation in these three regions. For each region representative single slices are shown whereas the fixation crosses show representative MNI-coordinates of single voxels (PCC: *x* = −1, *y* = −37, *z* = 30; CUN: *x* = 7, *y* = −97, *z* = 1; TPJ: *x* = −37, *y* = −65, *z* = 26). The images do not present standardized current density but relative differences in current density between orders. The shown single slices are representative of the ROIs which were statistically analyzed (see “Source Localization [Exact Low-Resolution Brain Electromagnetic Tomography Analyses (eLORETA)]—Affective Theory of Mind” in the results section).

Regarding the PCC, a highly significant main effect of order was shown, *F*_(2, 38)_ = 10.02, *p* = 0.00032 (^*^p = 0.0045). Inner subject contrasts revealed a significantly stronger activation in third-order trials (*M* = 0.028 μA/mm^2^, *SD* = 0.016) compared to first-order trials (*M* = 0.020 μA/mm^2^, *SD* = 0.015), *F*_(1, 19)_ = 14.68, *p* = 0.00113, and second-order trials (*M* = 0.023 μA/mm^2^, *SD* = 0.012), *F*_(1, 19)_ = 14.79, *p* = 0.00109. However, inner subject contrasts showed no difference between second-order and first-order trials, *F*_(1, 19)_ = 1.735, *p* = 0.203 (Figure 7).

Regarding the cuneus, a significant main effect of order was shown, *F*_(2, 38)_ = 7.3102, *p* = 0.0048 (^*^p = 0.0055). Inner subject contrasts indicated a stronger activation in third-order trials (*M* = 0.105 μA/mm^2^, *SD* = 0.08) compared to first-order trials (*M* = 0.072 μA/mm^2^, *SD* = 0.048), *F*_(1, 19)_ = 10.75, *p* = 0.003948, as well as stronger activation in second-order trials (*M* = 0.09 μA/mm^2^, *SD* = 0.061) compared to first-order trials, *F*_(1, 19)_ = 8.65, *p* = 0.0084. However, inner subject contrasts showed no difference between third-order and second-order trials, *F*_(1, 19)_ = 2.77, *p* = 0.112 (Figure 7).

Regarding the TPJ, a significant main effect of order was shown, *F*_(2, 38)_ = 6.848, *p* = 0.00288 (^*^p = 0.005). Inner subject contrasts showed a highly significantly stronger activation in third-order trials (*M* = 0.133 μA/mm^2^, *SD* = 0.134) compared to first-order (*M* = 0.089 μA/mm^2^, *SD* = 0.102), *F*_(1, 19)_ = 8.967, *p* = 0.000745, as well as a significantly stronger activation in third-order compared to second-order trials (*M* = 0.097 μA/mm^2^, *SD* = 0.087), *F*_(1, 19)_ = 7.61, *p* = 0.01249. However, inner subject contrasts showed no difference between second-order and first-order trials, *F*_(1, 19)_ = 0.7, *p* = 0.413 (Figure 7).

**Figure 8:**
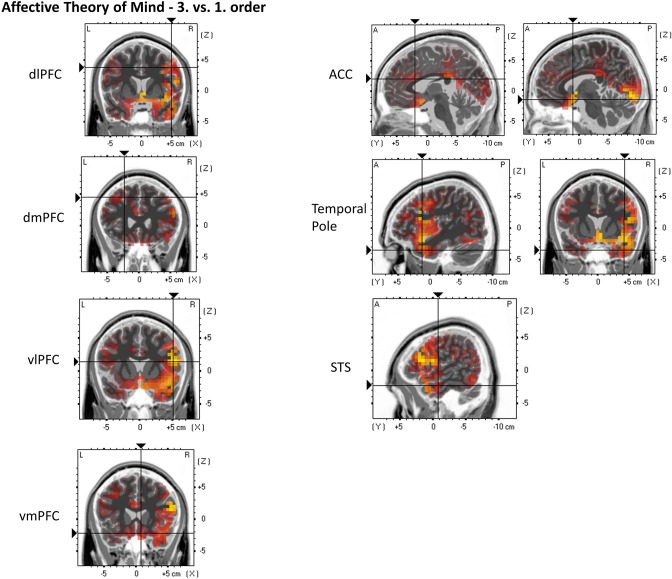
eLORETA source localization indicates differences in electrophysiological activity (current density, μA/mm^2^) between affective Theory of Mind third and first order trials in the dorsolateral prefrontal cortex (dIPFC), dorsomedial PFC (dmPFC), ventrolateral PFC (vIPFC), ventromedial PFC (vmPFC), Anterior Cingulate Cortex (ACC), Temporal Pole, and the Superior Temporal Sulcus (STS) within the time span of the Negative Slow Wave (NSW, 250–1,000 ms). The third order elicits the greater activation in these three regions. For each region representative single slices are shown whereas the fixation crosses show representative MNI-coordinates of single voxels [diPFC: *x* = 49, *y* = 7, *z* = 38; dmPFC: *x* = −23, *y* = 25, *z* = 44; viPFC: *x* = 52, *y* = 14, *z* = 14; vmPFC: *x* = 8, *y* = 20, *z* = −22; ACC: *x* = −3, *y* = 20, *z* = 21 (first image), *x* = 7, *y* = 10, *z* = −16 (second image); Temporal Pole: *x* = 43, *y* = 12, *z* = −35; STS: *x* = 56, *y* = −8, *z* = 22]. The images do not present standardized current density but relative differences in current density between orders. The shown single slices are representative of the ROIs which were statistically analyzed (see “Source Localization [Exact Low-Resolution Brain Electromagnetic Tomography Analyses (eLORETA)]—Affective Theory of Mind” in the results section).

For the following ROIs, no significant main or interaction effects could be found. Nevertheless, contrasts indicate greater activation in third-order affective ToM trials compared to first-order affective ToM trials: dlPFC (third: *M* = 0.052 μA/mm^2^, *SD* = 0.029 and first: *M* = 0.029 μA/mm^2^, *SD* = 0.018), *p* = 0.000174; dmPFC (third: *M* = 0.062 μA/mm^2^, *SD* = 0.051 and first: *M* = 0.034 μA/mm^2^, *SD* = 0.024), *p* = 0.01223; vlPFC (third: *M* = 0.065 μA/mm^2^, *SD* = 0.033 and first: *M* = 0.041 μA/mm^2^, *SD* = 0.026), *p* = 0.000038; vmPFC (third: *M* = 0.128 μA/mm^2^, *SD* = 0.094 and first: *M* = 0.085 μA/mm^2^, *SD* = 0.095), *p* = 0.0128; ACC (third: *M* = 0.03 μA/mm^2^, *SD* = 0.019 and first: *M* = 0.018 μA/mm^2^, *SD* = 0.015), *p* = 0.003549; precuneus (third: *M* = 0.188 μA/mm^2^, *SD* = 0.206 and first: *M* = 0.142 μA/mm^2^, *SD* = 0.141), *p* = 0.0412; TP (third: *M* = 0.092 μA/mm^2^, *SD* = 0.058 and first: *M* = 0.054 μA/mm^2^, *SD* = 0.039), *p* = 0.000023; and STS (third: *M* = 0.073 μA/mm^2^, *SD* = 0.037 and first: *M* = 0.05 μA/mm^2^, *SD* = 0.035), *p* = 0.000267. Furthermore, a stronger activation was indicated in second-order affective ToM trials compared to first-order affective ToM trials: STS (second: *M* = 0.076 μA/mm^2^, *SD* = 0.104 and first: *M* = 0.05 μA/mm^2^, *SD* = 0.035), *p* = 0.0084. For an overview, see Figure 8.

## Discussion

To date, only a few studies investigated the chronological aspects of ToM-specific brain activity, whereas, to the knowledge of the authors of the current study, none of these studies focused on higher order ToM reasoning. Therefore, the aim of the current study was to explore the electrophysiological correlates of basic and higher order ToM processing and its precise time course. This was operationalized by the ERP task “*Brainy-ERP*” that includes not only first- but also second- and third-order trials regarding both cognitive and affective ToM reasoning based on visual cues. This task was designed as a logical further development of the validated, established, and well-documented “Charlie” and “Yoni” ToM tasks (Baron-Cohen et al., 1995; Shamay-Tsoory and Aharon Peretz, 2007) and was adapted for ERP analyses. Based on the data recorded during *Brainy-ERP*, the electrophysiological activity of ToM processing was analyzed in the form of ERP components as well as activation of different brain areas, estimated by eLORETA. Another aim was to explore whether differences in behavioral scores and electrophysiological activity can be found between ToM low and high performers.

Behavioral results showed that participants reached high mean accuracies in all parts of *Brainy-ERP* (Table 1). Therefore, it can be assumed that all parts were equally understood which meant that no systematic bias had to be considered during ERP analyses. With respect to response time, on the other hand, it was shown that participants were slower in the first-order trials than in the second- and third-order trials (Table 1). Whereas, this seemingly contradicts expectations regarding difficulty of increasing orders, taking the results regarding accuracy into consideration, a training effect can be assumed. In this way, participants likely need more time during the first trials of each condition (cognitive and affective) in order to understand the requirements of the task. Results further showed medium to high correlations between cognitive and affective ToM with respect to behavioral data (Table 2).

In the course of analyzing the EEG data, different ERP components were identified upon visual inspection (see the Electroencephalography—Event-Related Potentials subsection of the Methods section). With respect to these components, it was further explored whether visual differences between orders could be detected. Visual analyses yielded no differences between orders with respect to “classic” visual components. These are typically influenced by visual complexity such as basic visual stimulus parameters as well as by selective attention or are associated with discrimination tasks (see e.g., Luck, 2014). The absence of differences in initial (visual) processing between orders is further supported by task-specific stimulus properties. Stimuli were of similar size and color, fixation crosses always indicated the position where the relevant objects appeared (object, smiley, Brainy), objects appeared in close proximity, and objects always appeared in fixed positions (e.g., Brainy appeared always in the middle of the slide). See the Stimuli and Task section.

The first statistical significant difference between orders was shown for the bilateral anterior P2 component (180–370 ms p.s.) in both cognitive and affective trials. It was shown that in both conditions, the first-order trials elicited greater P2 amplitudes than the second- or third-order trials. Previously, the anterior P2 has been associated with higher order perceptual and attentional processing, specifically regarding visual input analysis (e.g., Luck and Hillyard, 1994) as well as with top-down matching processes, as perceptual information is compared to contextually derived expectations (Federmeier et al., 2005). Given the result that basic ToM elicited greater P2 amplitudes than higher order ToM, it can again be argued that within conditions, a training effect took place. In this way, it is conceivable that the first trials of each condition required more effort to interpret the visual input and to evaluate it in the light of the previously shown statement. Therefore, it can be hypothesized that the process of identifying target features (see e.g., Luck, 2014) becomes more automatized in later, higher order trials.

In cognitive trials, differences between orders were shown regarding a negative deflection at 350–470 ms p.s. which was named the early negative slow wave. This component was broadly distributed at bilateral frontal, temporal, and parietal sites. It was shown that third-order trials yielded greater eNSW amplitudes than second- and first-order trials, whereas the latter did not differ (Figure 3). This component shows similarities to a frontal slow wave potential described by Kühn-Popp et al. (2013) which was more negative during pretense reasoning than during false belief reasoning. In the said study, this slow wave was associated with attributing intentions and other mental states to a protagonist in order to explain a presented scenario, similar to the attribution of beliefs and thoughts to the protagonists during *Brainy-ERP*. Furthermore, in previous studies, frontal NSWs were associated with conceptual memory processing (Lang et al., 1987). In the current study, participants had to memorize a statement preceding each trial involving the memorization of sequential visual cues so as to correctly respond at the end of each trial. Supporting the assumption that statements of increasing complexity—as they involve an increasing number of recursive steps as well as protagonists—should lead to a higher cognitive load, it was shown in the current study that negative mean amplitudes increased with order. In the current study, the sites of the eNSW included parietal and temporoparietal electrodes. According to Ruchkin et al. (1992), posterior NSWs are associated with visuospatial working memory. Since the position of the protagonists on the last slide of each trial in combination with their eye gazes is essential to deduce their respective thoughts and beliefs, an association with this previously discovered posterior slow wave seems reasonable. Supporting this notion, previous studies indicated an association between visual–spatial mentalization/visual perspective taking and ToM processing (e.g., Gabriel et al., 2019) and shared neuronal activation regarding these abilities as for example in the left TPJ, the PCC, and the precuneus (Schurz et al., 2013).

In the time span of the cognitive eNSW (350–470 ms p.s.), only a statistical trend was shown with respect to differences in activity in underlying brain regions. Analyses indicated that second- and third-order trials elicit a greater activation than first-order trials in the PCC. The PCC is part of the previously described ToM network (see Abu-Akel and Shamay-Tsoory, 2011) and is associated with self-mental states (Lou et al., 2004). Furthermore, Leech and Sharp (2013) suggested that the dorsal PCC may influence attentional focus by adjusting the stability of brain network activity over time.

Furthermore, in cognitive trials, differences between orders were shown regarding a negative deflection at 460–1,000 ms p.s. which was named the late negative slow wave. This component was broadly distributed at left-hemispheric frontal, temporal, and parietal sites as well as at frontal sites on the right hemisphere. It was shown that third-order trials yielded greater LNSW amplitudes than second- and first-order trials, whereas the latter did not differ (Figure 3). In previous studies (Meinhardt et al., 2011; Kühn-Popp et al., 2013), posterior LSWs were associated with the integration of visual stimuli into an internal representation of another individual's mental state. A similar process would explain the increasing amplitude of the waveforms with respect to the order of ToM reasoning based on visual cues as the number of protagonists and eye gazes included in the tasks increases as well. However, the late frontal activity may reflect the last stage of an initial ToM processing; the resulting attribution of a belief or thought to another individual after taking all visual and conceptual information into account. This undertaking may require more information for second- and third-order than for first-order processing, resulting in more negative amplitudes at frontal sites. Results further showed medium to high correlations between mean amplitudes of cognitive and affective ToM components (Table 2). This result is supported by studies that show overlapping activation in cognitive and affective ToM networks (see e.g., Abu-Akel and Shamay-Tsoory, 2011).

In the time span of the cognitive LNSW (460–1,000 ms p.s.), again, only a statistical trend was shown with respect to differences between orders in the PCC. Analyses indicated that third-order trials elicit a greater activation than first-order trials. With respect to the absence of statistical differences between orders regarding the previously mentioned ROIs, it can be hypothesized that within regions of the ToM network, only specific subregions showed increased activity so that overall activity in the region was too small to reach statistical significance. It can be further hypothesized that the presence of only one ToM-specific cue (eye gaze) in the cognitive condition as well as the previously suggested training effect could possibly lead to such a situation in which only specific areas of the ROIs contribute to ToM network activation across orders. Based on these results, the authors of the study inspected the eLORETA visualization of the differences regarding current density between the third and the first order in the LNSW time span in the course of which differences in right-hemispheric prefrontal and frontal regions are noticeable. Nevertheless, as previously stated, they did not reach statistical significance. This right-hemispheric prefrontal and frontal activation is probably involved in the asymmetrical site distribution of the LNSW (Figure 3).

In contrast to cognitive trials, in affective trials, differences between orders were shown regarding a long-lasting negative deflection at 250–1,000 ms p.s. which was named the negative slow wave. This component was broadly distributed at bilateral frontal, temporal, and parietal sites. It was shown that third-order and second-order trials yielded greater NSW amplitudes than first-order trials (see Figure 4). In comparison to the two consecutive slow waves in the cognitive condition, only in affective trials such a long-lasting NSW could be seen. Besides a number of parallels between cognitive and affective ToM processing within the current paradigm, the most prominent difference lies therein that the processing of affective ToM trials is associated with an additional ToM-specific cue in the form of the protagonists' facially expressed emotions (smiling/unhappy expression).

The differences at frontal sites can be possibly explained in a similar way as earlier with respect to the cognitive trials; they are likely associated with the attribution of mental states (e.g., Meinhardt et al., 2011). In this context, the differences in dlPFC, dmPFC, vlPFC, vmPFC, and ACC activation, as indicated by eLORETA (Figure 8), would theoretically support this interpretation as it was previously associated with determining behavior based on anticipation (see e.g., Amodio and Frith, 2006). Differences at the frontal sites of the NSW component can further be, at least partially, supported by studies on the integration of neutral and emotional stimulus properties. In this line, Yick et al. (2015) linked a long-lasting anterior activity (400–1,000 ms p.s.) to the elaboration of a stimulus' semantic meaning which was greater when correctly retrieving emotional information compared to neutral information.

At posterior sites, the integration of additional visual information into the participant's inner representation of the protagonists' beliefs and emotional states (see e.g., Kühn-Popp et al., 2013) may be reflected by the increased amplitude of both second- and third-order trials compared to first-order trials. On the other hand, differences between participants regarding the order in which the visual cues (eye gaze/expression) are processed might (additionally) cause such a broadly distributed activity. Again, these results are in line with the study of Yick et al. (2015) which show a long-lasting posterior activity which they linked to processing and integration of sensory information related to attended stimuli. This component, again, showed higher amplitudes when emotional information was retrieved correctly compared to neutral information. This integration of semantic and perceptual processing would be in line with the multimodal features of emotional information (Yick et al., 2015). The current results showing increased amplitudes at both hemispheres can potentially be linked to the results of the eLORETA analyses which showed that third-order trials consistently elicited the strongest activation in the PCC, the cuneus, as well as the TPJ, all regions of the established ToM network (see e.g., Abu-Akel and Shamay-Tsoory, 2011). The cuneus and the TPJ were previously associated with processing others' mental states (e.g., Saxe and Kanwisher, 2003; Schlaffke et al., 2015; Lin et al., 2018; Boccadoro et al., 2019; Mukerji et al., 2019), whereas the TPJ was additionally associated with other cognitive functions such as attention, memory, and language processing (e.g., Igelström et al., 2015). The PCC together with the precuneus, on the other hand, was previously associated with processing mental states of oneself (Lou et al., 2004). It can be hypothesized that in higher order affective ToM, stronger activation in these regions is required as mental states of multiple protagonists including different ToM-specific cues (eye gazes, expressions) need to be taken into consideration in order to fulfill the task.

As another aim of the current study was to investigate differences between ToM high and low performers, the sample was divided into the fastest 25% (ToM high performer, “fast responder”), middle 50% (ToM average performer, “average responder”), and the slowest 25% (ToM low performer, “slow responder”) based on response time. No performance groups were built on total accuracy percentage due to low variation in accuracy scores with high scores across all parts of *Brainy-ERP* (Table 1). Please note that the following results are explorative due to the small sample size of the performer groups. Behavioral results regarding performer groups showed no significant differences between groups regarding response accuracy percentage, again indicating a ceiling effect regarding performance. Nevertheless, regarding differences between performer groups with respect to ERP components, a statistical trend was shown regarding the cognitive second-order ENSW component (260–470 ms p.s.) on the right hemisphere. Results indicated that fast responders showed greater mean amplitudes than slow responders, whereas average responders showed intermediate values. Furthermore, with respect to cognitive third-order posterior N1 component (120–250 ms p.s.), fast responders and average responders did not differ but both showed significantly greater mean amplitudes than slow responders. Please see Figure 6 and note that cognitive second-order trials were performed before cognitive third-order trials. Regarding these results, it can be hypothesized that in the earlier occurring second-order trials, slow responders show a slight disadvantage in the attribution of mental states to the protagonists, whereas this ability is seemingly associated with conceptual memory processing (see e.g., Lang et al., 1987; Kühn-Popp et al., 2013). With regard to the previous discussion on the role of the eNSW, it is conceivable that slow responders have at least initial difficulties coping with the memorization of an increasing number of sequential visual cues as well as analyzing an increasing number of recursive steps. Subsequently, slow responders show at cognitive third-order trials significantly smaller mean amplitudes regarding an ERP component that was previously associated with discrimination tasks and was shown to be influenced by spatial attention (see e.g., Luck, 2014). Therefore, it can be hypothesized that individuals who show at least average response times benefit more from preceding trials that are similar and are therefore more capable to effectively use visual information in earlier stages of ToM processing. This hypothesis would at least be partially supported by structural and functional overlaps between ToM and attention brain networks (see e.g., Abu-Akel and Shamay-Tsoory, 2011; Koziol et al., 2014). The results that average and fast responders did not differ significantly, and that for affective trials, no differences were shown, could be possibly explained by the previously mentioned possible ceiling and training effects in a way that differences between performer groups decrease throughout the task.

## Conclusion

The current study explored the electrophysiological correlates of basic and higher order ToM processing and its precise time course in the form of ERP components as well as activation of different brain areas, estimated by eLORETA. It was shown that basic order cognitive and affective ToM processing elicited greater anterior P2 amplitudes than higher order ToM processing. This was interpreted in a way that the first trials of each condition (cognitive and affective) required more effort to analyze the visual input with respect to ToM-specific target features and to integrate this information. It was suggested that this process becomes more automatized in subsequent higher order ToM trials. With respect to cognitive ToM processing, two ERP components were identified for which differences between basic and higher order ToM were shown, namely, an early negative slow wave and a late negative slow wave, both broadly distributed across frontal, temporal, and parietal sites. Higher order cognitive ToM in the form of third-order trials showed the greatest eNSW amplitudes, whereas similar components were previously associated with ToM-specific attribution of mental states to others as well as with conceptual memory processing and visual–spatial working memory. In case of the current task, it was interpreted that higher order ToM requires more effort to memorize multiple sequential task-relevant visual cues as well as to integrate them in the course of ToM processing. Higher order cognitive ToM trials in the form of third-order trials further elicited the greatest LNSW amplitudes, whereas similar components were previously associated with the integration of visual stimuli into an internal representation of another individual's mental state. In case of the current task, it was interpreted that higher order ToM requires more effort to integrate information into such internal representations as higher order ToM involves a greater number of protagonists and ToM-specific visual cues to take into account. With respect to affective ToM processing, a long-lasting NSW was shown that was broadly distributed across frontal, temporal, and parietal sites. Higher order ToM in the form of second- and third-order trials elicited greater NSW amplitudes than basic ToM trials. Interestingly, this long-lasting slow wave covers approximately the same time span as the two consecutive slow waves in the cognitive condition. It was hypothesized that the affective NSW has underlying processes similar to cognitive ToM, whereas the additional ToM-specific cue (adding emotional expressions) as well as differences between participants in which order these visual cues are processed may lead to such a long-lasting component instead of two distinct components. It was interpreted in a way that greater amplitudes in higher order ToM trials can on the one hand be linked to increased attribution of mental states which was previously associated with greater amplitudes at frontal sites. This could be supported by further results of the current study which indicated increased activation of prefrontal and limbic regions in affective higher order trials with respect to the time span of the NSW. On the other hand, greater amplitudes in higher order ToM were linked to an increased effort to integrate additional visual information into the participant's inner representation of the protagonists' mental states which was previously associated with greater amplitudes at posterior sites. This could be supported by further findings of the current study which indicated increased activation of the PCC, the cuneus, and the TPJ in affective higher order trials which was estimated by eLORETA. These regions were previously associated with representations of mental states of oneself and others. The current study further aimed to investigate differences between ToM high and low performers. Whereas, ToM slow, average, and fast responders did not differ regarding behavioral accuracy, differences as well as statistical trends were shown regarding cognitive second-order eNSW and consecutively regarding cognitive third-order Posterior N1 mean amplitudes. In the earlier occurring second-order trials, slow responders tended to show smaller amplitudes compared to average and fast responders regarding the eNSW. This was interpreted in a way that slow responders probably show slight disadvantages in conceptual memory processing and at least initial difficulties coping with the memorization of an increasing number of sequential visual cues as well as analyzing an increasing number of recursive steps at this stage of processing. Subsequently, in the later occurring third-order trials, slow responders showed significantly lower mean amplitudes compared to average and fast responders regarding the posterior N1 component which was previously associated with discrimination tasks and spatial attention. This was interpreted in a way that individuals who show at least average response times potentially benefit more from preceding trials that are similar and are therefore more capable to effectively use visual information in earlier stages of ToM processing.

## Limitations

The first limitation of the current task is that although *Brainy-ERP* is a further development of established ToM tasks, it nevertheless is a task that is based on comic images which reduce its meaningfulness with respect to real-life everyday social interaction to a certain degree. This does probably apply more to the affective ToM trials as only smiling or frowning expressions were presented, not accounting for the variety of everyday emotional expressions as well as subtle differences regarding those. Nevertheless, the current study offers new insights into the electrophysiological basis of basic and higher order ToM processing.

The second limitation of the study is the small sample size. Nevertheless, the ToM task comprised a great amount of presented stimuli which to some degree should compensate for the sample size. Nevertheless, the sample sizes of the performer groups are very small. Please note that statistical analyses regarding those groups are explorative.

The third limitation of the study is that the sample solely consists of high-performing medical students. Whereas, this sample is hardly comparable to samples used in previous studies that featured similar ToM tasks, such as underage or psychiatric individuals, the current study shows results that are with respect to basic ToM comparable to previous ToM ERP studies and offers new insights into electrophysiological activity regarding higher order ToM processing.

The fourth limitation is that the order of conditions was not randomized across participants which possibly elicited training effects regarding both behavioral scores and electrophysiological activity. Future studies are needed to investigate such effects.

Furthermore, the results regarding source localization need to be treated with caution. Despite being a validated method, it nevertheless represents a calculated estimation of the activation of specific brain regions (for details, see the Methods section).

## Data Availability Statement

The datasets generated for this study are available on request to the corresponding author.

## Ethics Statement

Before participation, all participants gave their written informed consent. This study protocol was approved by the Institutional Review Board of the respective University and meets the ethical principles of the Declaration of Helsinki as well as the APA ethical standards for human research.

## Author Contributions

UW, MS, MD, and BT conceptualized and designed the study and adapted the task for ERP recordings. BT and MD collected the data, conducted analyses, and drafted and revised the initial manuscript. All authors contributed to manuscript revision and read and approved the submitted versions.

### Conflict of Interest

The authors declare that the research was conducted in the absence of any commercial or financial relationships that could be construed as a potential conflict of interest.

## Footnotes

^1^Psychology Software Tools Inc. [E-Prime 2.0]. Retrieved from https://www.pstnet.com.

## Abbreviations

ACC: anterior cingulate cortex
Brainy-ERP: event-related potentials version of the Brainy task
dlPFC: dorsolateral prefrontal cortex
dmPFC: dorsomedial prefrontal cortex
EEG: electroencephalography
ERP: electroencephalographic event-related potential
eNSW: early negative slow wave
fMRI: functional magnetic resonance imaging
LORETA: low-resolution electromagnetic tomography analyses
LPC: late positive component
LNSW: late negative slow wave
NSW: negative slow wave
PCC: posterior cingulate cortex
PFC: prefrontal cortex
rmANOVA: repeated-measures analysis of variance
STS: superior temporal sulcus
ToM: Theory of Mind
TP: temporal pole
TPJ: temporoparietal junction
vlPFC: ventrolateral prefrontal cortex
vmPFC: ventromedial prefrontal cortex.
